# Reduced Bacterial Colony Count of Anaerobic Bacteria Is Associated with a Worsening in Lung Clearance Index and Inflammation in Cystic Fibrosis

**DOI:** 10.1371/journal.pone.0126980

**Published:** 2015-05-20

**Authors:** Katherine O’Neill, Judy M. Bradley, Elinor Johnston, Stephanie McGrath, Leanne McIlreavey, Stephen Rowan, Alastair Reid, Ian Bradbury, Gisli Einarsson, J. Stuart Elborn, Michael M. Tunney

**Affiliations:** 1 CF and Airways Microbiology Research Group, Queen’s University Belfast, Belfast, United Kingdom; 2 Centre for Infection and Immunity, School of Medicine, Dentistry and Biomedical Sciences, Queen’s University Belfast, Belfast, United Kingdom; 3 Centre for Health and Rehabilitation Technologies (CHART), University of Ulster, Belfast, United Kingdom; 4 School of Pharmacy, Queen’s University Belfast, Belfast, United Kingdom; 5 Belfast Health and Social Care Trust, Belfast, United Kingdom; University of Dundee, UNITED KINGDOM

## Abstract

Anaerobic bacteria have been identified in abundance in the airways of cystic fibrosis (CF) subjects. The impact their presence and abundance has on lung function and inflammation is unclear. The aim of this study was to investigate the relationship between the colony count of aerobic and anaerobic bacteria, lung clearance index (LCI), spirometry and C-Reactive Protein (CRP) in patients with CF. Sputum and blood were collected from CF patients at a single cross-sectional visit when clinically stable. Community composition and bacterial colony counts were analysed using extended aerobic and anaerobic culture. Patients completed spirometry and a multiple breath washout (MBW) test to obtain LCI. An inverse correlation between colony count of aerobic bacteria (n = 41, r = -0.35; p = 0.02), anaerobic bacteria (n = 41, r = -0.44, p = 0.004) and LCI was observed. There was an inverse correlation between colony count of anaerobic bacteria and CRP (n = 25, r = -0.44, p = 0.03) only. The results of this study demonstrate that a lower colony count of aerobic and anaerobic bacteria correlated with a worse LCI. A lower colony count of anaerobic bacteria also correlated with higher CRP levels. These results indicate that lower abundance of aerobic and anaerobic bacteria may reflect microbiota disruption and disease progression in the CF lung.

## Introduction

Cystic Fibrosis (CF) lung disease is characterised by inflammation, recurring and chronic infection causing lung injury, progressive loss of lung function and eventual respiratory failure. Bacterial species such as *Pseudomonas aeruginosa*, *Staphylococcus aureus* and *Burkholderia cepacia* complex (Bcc) are considered to be the predominant pathogens responsible for CF lung infection [1–3]. However, extended culture and molecular detection methods have demonstrated that there is much greater bacterial diversity in the CF lung than previously thought, with aerobic and anaerobic bacteria, fungi and viruses all contributing to a complex airway microbiota [4–7]. Anaerobic bacteria have been identified in abundance in a wide age range of CF patients [5,6]; however, their clinical relevance and specific role, if any, in pulmonary infection and inflammation requires further study.

The presence of anaerobic bacteria not previously detected by culture may be an explanation as to why some CF patients have evidence of pulmonary inflammation in the absence of known pathogens or why some patients fail to respond clinically to antibiotic treatment [8,9]. Anaerobic species such as *Prevotella*, possess known virulence factors and may facilitate and create a favourable environment for other bacterial organisms to colonise [10]. Conversely, direct evidence of a correlation between the presence and/or abundance of anaerobic bacteria and lung disease progression in CF is lacking [11] and it may be possible that these bacteria constitute part of the healthy lung microbiota where disruption may lead to disease, as suggested in studies of COPD and asthma [12,13]. Study of the association between the abundance of anaerobic bacteria and lung disease severity in CF is required to help determine if anaerobic bacteria contribute to the pathogenesis of CF lung disease.

Lung Clearance Index (LCI) derived from Multiple Breath Washout (MBW) is a robust surrogate outcome measure of lung disease in CF [14] and was the primary measure of interest in this study. LCI measures ventilation inhomogeneity, i.e. uneven gas mixing throughout the lungs which has been shown to be more sensitive to lung function abnormality than FEV_1_% predicted [15], a discriminator of airway infection in patients with *P*. *aeruginosa* and *S*. *aureus* [16,17] and a predictor of time to first pulmonary exacerbation (PEx).[18]

The aim of this study was to assess the relationship between microbial colony count (aerobic and anaerobic bacteria) and community composition and (i) LCI (ii) spirometry and (iii) C—reactive protein (CRP); in patients with CF. Data from healthy controls provided a comparator group.

## Methods

### Patients

Adults and children (aged ≥ 6 years), with a confirmed diagnosis of CF [19] who were clinically stable (no PEx requiring intravenous antibiotics [IVAB] in the previous 4 weeks) were recruited from outpatient clinics at the adult and paediatric CF Centres at Belfast Health and Social Care Trust (BHSCT) and data was collected at a single cross-sectional visit. Patients with Bcc infection were excluded due to issues relating to infection control with MBW equipment.

Healthy control patients (aged ≥ 18years) were recruited from staff groups at Queen’s University Belfast (QUB) and BHSCT. Exclusion criteria were (1) diagnosis of a respiratory condition, (2) use of respiratory medication or antibiotics in the last 4 weeks, (3) current smoker or (4) participating in an investigational clinical trial in the previous 4 weeks.

### Sputum collection and bacterial isolation and identification

Expectorated and induced sputum samples were collected from CF patients and healthy subjects, respectively. Control subjects performed an induced sputum procedure using 7% hypertonic saline and an Omron MicroAIR NE-U22-E nebuliser. Subjects were instructed to take 3–4 tidal breaths interspersed with 1–2 deep breaths and stop the inhalation every 1 to 2 minutes in order to cough up sputum. Saliva and sputum was collected in the same container. This cycle was continued as tolerated until an adequate volume of sputum (>1g) was collected. Samples were immediately placed in an anaerobic pouch and transported to the laboratory for processing in an anaerobic cabinet. Strict aerobic and anaerobic bacteriological culture techniques were used to provide an in-depth analysis of the microbiota composition and abundance of aerobic and anaerobic bacteria. Culture and subsequent detection of isolates in samples was performed as detailed in S1 File and as previously described [5,6] with all bacteria detected quantified (colony forming units/gram sputum; [cfu/g]) by total viable count (TVC) and identified by PCR and sequencing of 16S ribosomal genes.

### Clinical data

Demographic data were recorded from CF patient’s notes. Infection with *P*. *aeruginosa* was defined using the Leeds criteria [20]. High sensitivity blood CRP was measured by turbidimetric immunoassay in QUB laboratories, from a blood sample taken on the same day.

### MBW test

The MBW test to measure LCI was carried out using the modified Innocor device and 0.2% sulfur hexafluoride (SF_6_) using the open-circuit technique in accordance with the standard operating procedure developed by the UK CF Gene Therapy Consortium (UKCFGTC) (S2 File). This technique was previously validated by Horsley et al.[15] and replicated at the Belfast site [21] Furthermore, it has been incorporated into CF clinical trials [22,23]. Patients breathed through a mouthpiece at normal tidal volumes, whilst in a seated position and wearing a nose clip. Analysis of MBW data was performed using the Simple Washout programme developed by Dr Nicholas Bell (UKCFGTC) and used with his permission. LCI was calculated from a minimum of 2 technically valid and repeatable tests (S2 File).

### Spirometry

Spirometry was measured according to ATS/ERS guidelines [24] using a Microlab (ML3500 MK8) spirometer (CareFusion, Kent, UK). Predicted values and z-scores were calculated from reference ranges for all ages [25].

### Data analysis

No formal sample size calculation was carried out. Data were analysed using PASW Statistics (version 18, IBM software, USA) and Prism (Version 5.01 GraphPad Software Inc.) packages. Subject characteristics were summarised with standard descriptive statistics. Colony count of aerobic and anaerobic bacteria and blood CRP were log_10_ transformed for statistical analysis. Wilcoxon signed rank tests were used to assess for differences between the colony count of aerobic and anaerobic bacteria in samples from CF patients and control subjects. A Spearman’s rank correlation coefficient was used to measure the strength of the linear association between LCI, FEV_1_ z-score and colony counts (aerobic, anaerobic bacteria) and between colony count (aerobic, anaerobic bacteria) and CRP. Multiple linear regression analysis was used to assess the contribution of potential confounding factors in predicting colony counts. A *p*-value of <0.05 was considered statistically significant. This study was approved by the Office for Research Ethics Committees Northern Ireland (ORECNI) (REC reference number: 10/NIR01/41) and co-sponsored by BHSCT and QUB (research office reference number: 10067SE-OPMS). Patients or their parents provided written informed consent as approved by ORECNI.

## Results

### Patients

Sixty-three CF patients and 17 healthy control subjects were enrolled and attempted the study procedures at a single study visit. The induced sputum procedure was attempted in control subjects only in this study and was not attempted in CF patients due to lack of time (study visits were conducted as part of a routine outpatient appointment). Only a small number of healthy control subjects (n = 6; 35%) produced an adequate volume of induced sputum. Forty-one (65%) CF patients produced adequate volumes of sputum for culture analysis (S1 Fig). Therefore, full data was analysed for 41 CF patients and 6 healthy control subjects.

In the 41 CF patient and 6 control subjects included in analysis, there was no difference in age, sex or LCI CV% between the 2 groups. However, there were significant differences in FEV_1_% predicted, FEV_1_ z-score and LCI (Table 1).

**Table 1:** CF patient and healthy control subject characteristics.

	CF n = 41	Control n = 6	*p*-value
Mean (SD) age [range] (years)	29.2 (14.0) []8–67	34.0 (9.0) [24–44]	0.28
Females:males (n)	15:26	4:2	0.16
N (%) F508del homozygous	12/41 (29)	n/a	n/a
N (%) chronic *P*. *aeruginosa*	20/41 (49)	n/a	n/a
Mean (SD) FEV_1_% predicted	71.2 (16.6)	101.3 (11.8)	<0.001
Mean (SD) FEV_1_ z-score	-2.4 (1.3)	0.1 (1.0)	<0.001
Mean (SD) LCI [range] (no. turnovers)	10.6 (2.6) [5.4–16.4]	6.4 (0.4) [5.8–7.0]	<0.001
Mean (SD) LCI coefficient of variation (CV) %	4.4 (2.6)	3.4 (2.0)	0.44
N (%) anti-pseudomonal antibiotic^a^	24/41 (59)	n/a	n/a
N (%) oral azithromycin	23/41 (56)	n/a	n/a

^a^Oral and/or inhaled anti-pseudomonal

### Culture analysis of lung microbiota composition in CF patients and control subjects

**Fig 1:**
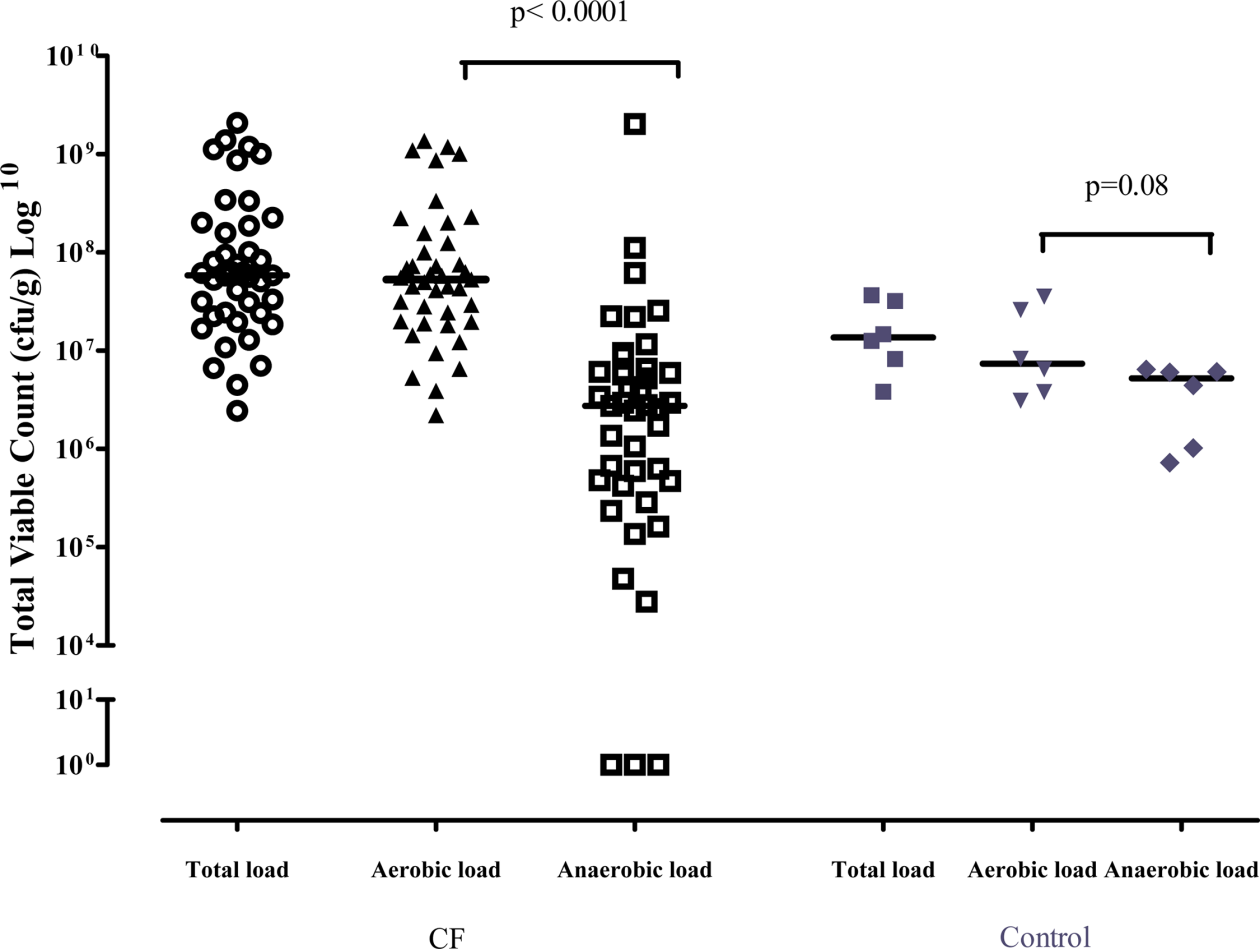
Total colony count, aerobic colony count and anaerobic colony count of bacteria in sputum from CF patients and control subjects. (Median).

In patients with CF, bacteria were detected in high abundance (up to 2.1 x 10^9^ cfu/g) in the 41 sputum samples. A mean (SD) [range] of 6.7 (1.8) [3–11] different genera were cultured per sample with a median (IQR) colony count of 5.8 x 10^7^ (2.3 x 10^7–^1.9 x 10^8^) cfu/g sputum. Aerobic bacteria from 25 different genera were isolated in high abundance (up to 1.4 x 10^9^ cfu/g) in all samples with *Streptococcus* (40/41 patients), *Rothia* (29/41 patients), *Staphylococcus* (20/41 patients), *Actinomyces* (20/41), *Pseudomonas* (20/41 patients), *Gemella* (17/41 patients), the predominant genera detected. Anaerobic bacteria from 12 different genera were also detected in high numbers (up to 2.0 x 10^9^ cfu/g) from 38 of 41 samples (93%), with *Prevotella* (34/41 patients) and *Veillonella* (22/41 patients) most frequently isolated. Aerobic bacteria were present in significantly greater numbers than anaerobic bacteria (median difference in colony count 5.0 x 10^7^ cfu/g; Wilcoxon signed rank test, p<0.0001) (Fig 1).

In the 6 induced sputum samples from control patients, bacteria were also detected in abundance (up to 3.7 x 10^7^ cfu/g). A mean (SD) [range] of 8.3 (1.5) [7–11] different genera were cultured per sample with a median (IQR) colony count of 1.4 x 10^7^ (7.1 x 10^6–^3.3 x 10^7^) cfu/g sputum. Aerobic bacteria from 10 different genera were isolated in high abundance (up to 1.4 x 10^7^ cfu/g) in all samples with *Streptococcus* (6/6), *Haemophilus* (6/6) and *Rothia* (6/6), most frequently isolated. Anaerobic bacteria from 3 different genera were also detected in high numbers (up to 6.4 x 10^6^ cfu/g) in all samples, with *Prevotella* (6/6 patients) and *Veillonella* (3/6 patients) most frequently detected. There was no significant difference between colony count of aerobic and anaerobic bacteria (median difference in colony count 2.1 x 10^6^ cfu/g; Wilcoxon signed rank test, p = 0.08) (Fig 1).

### Relationship between lung function and microbial colony counts

**Fig 2:**
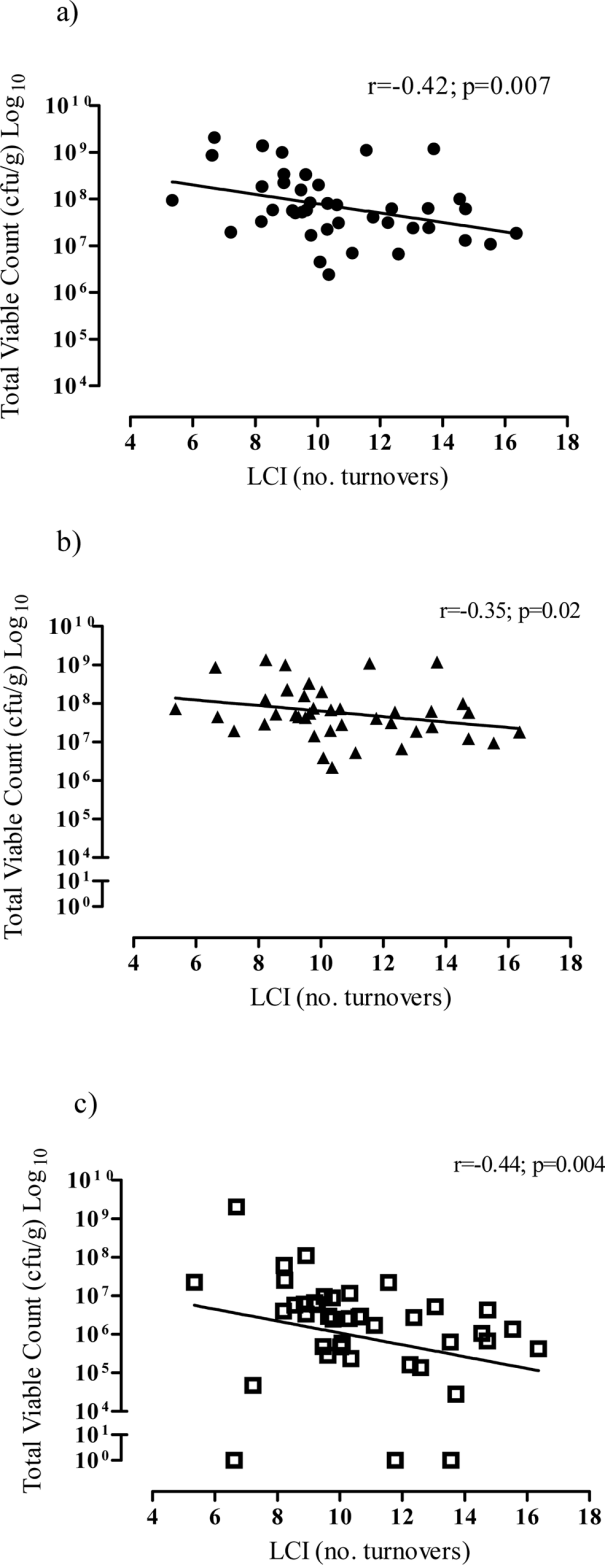
Relationship between LCI and a) total colony count b) aerobic colony count and c) anaerobic colony count.

In CF samples, there was a significant inverse correlation between total colony count and LCI (r = -0.42; p = 0.007) (Fig 2A). Considering aerobic and anaerobic bacteria separately, there was a significant inverse correlation between LCI and colony count of aerobic bacteria (r = -0.35; p = 0.02) and anaerobic bacteria (r = -0.44; p = 0.004), indicating that a lower colony count was associated with a higher i.e. worse LCI. (Fig 2B and 2C).

**Fig 3:**
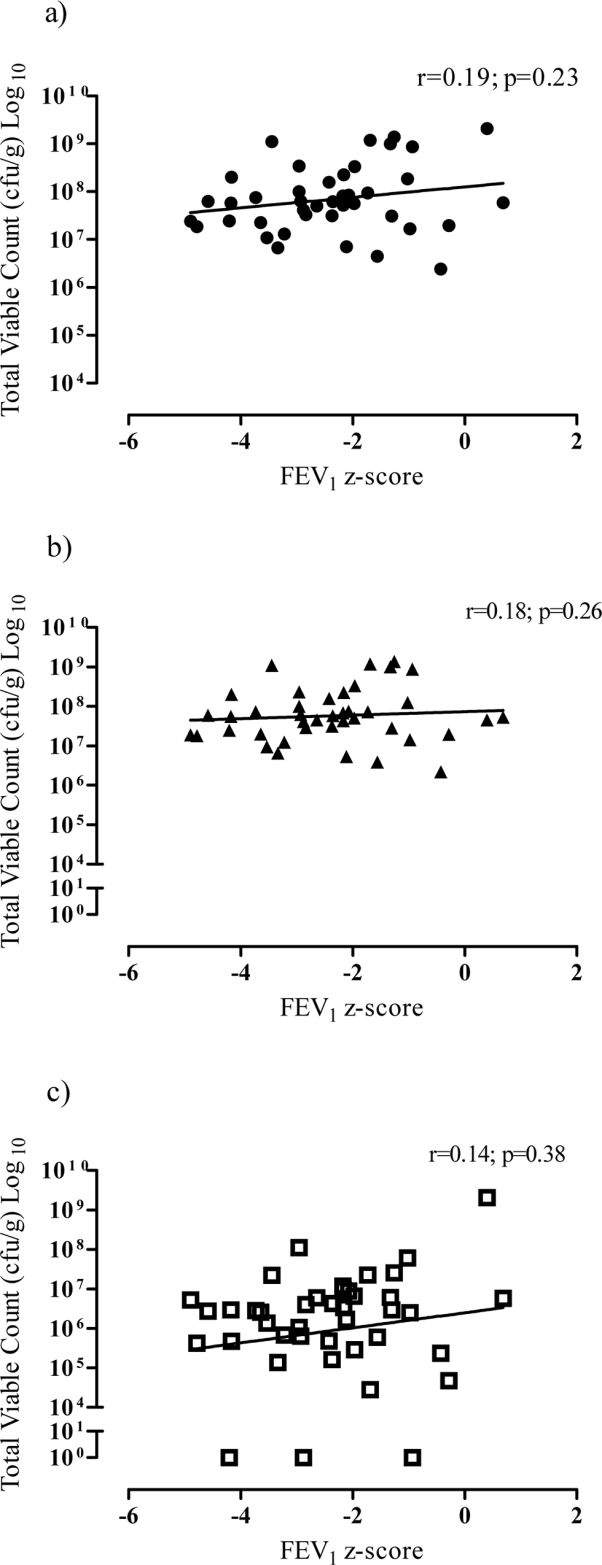
Relationship between FEV_1_ z-score and a) total colony count b) aerobic colony count and c) anaerobic colony count.

The relationships between FEV_1_ z-score and total colony count (r = 0.19; p = 0.23), colony count of aerobic (r = 0.18; p = 0.26) and anaerobic (r = 0.14; p = 0.38) bacteria in CF samples were not significant (Fig 3A–3C). Similarly, the relationship between FEF_25–75_ z-score and total colony count (r = 0.26; p = 0.12), colony count of aerobic (r = 0.18; p = 0.29) and anaerobic (r = 0.27; p = 0.10) bacteria in CF samples were not significant.

Considering other factors that could potentially impact results, there was no correlation between age and aerobic (r = -0.19; p = 0.24) or anaerobic colony counts (r = 0.15; p = 0.35). Furthermore, no anaerobic bacteria were detected by culture in 3 samples; when excluded from analysis, the correlation between LCI and anaerobic colony count remained statistically significant (r = -0.50; p = 0.001) and the correlation between FEV_1_ z-score and colony count of anaerobic bacteria remained not significant (r = 0.16; p = 0.35).

As *Prevotella* and *Veillonella* were the most abundant anaerobic organisms detected in CF samples in this study (82% and 54% of samples, respectively), we assessed the relationship between these genera and lung disease severity. An inverse correlation was observed between abundance of *Veillonella*, LCI (r = -0.80; p<0.0001) and FEV_1_ z-score (r = 0.64; p = 0.001) (S2 Fig). However, a relationship was not evident between the abundance of *Prevotella*, LCI (r = -0.12; p = 0.50) or FEV_1_ z-score (r = -0.06; p = 0.74).

**Fig 4:**
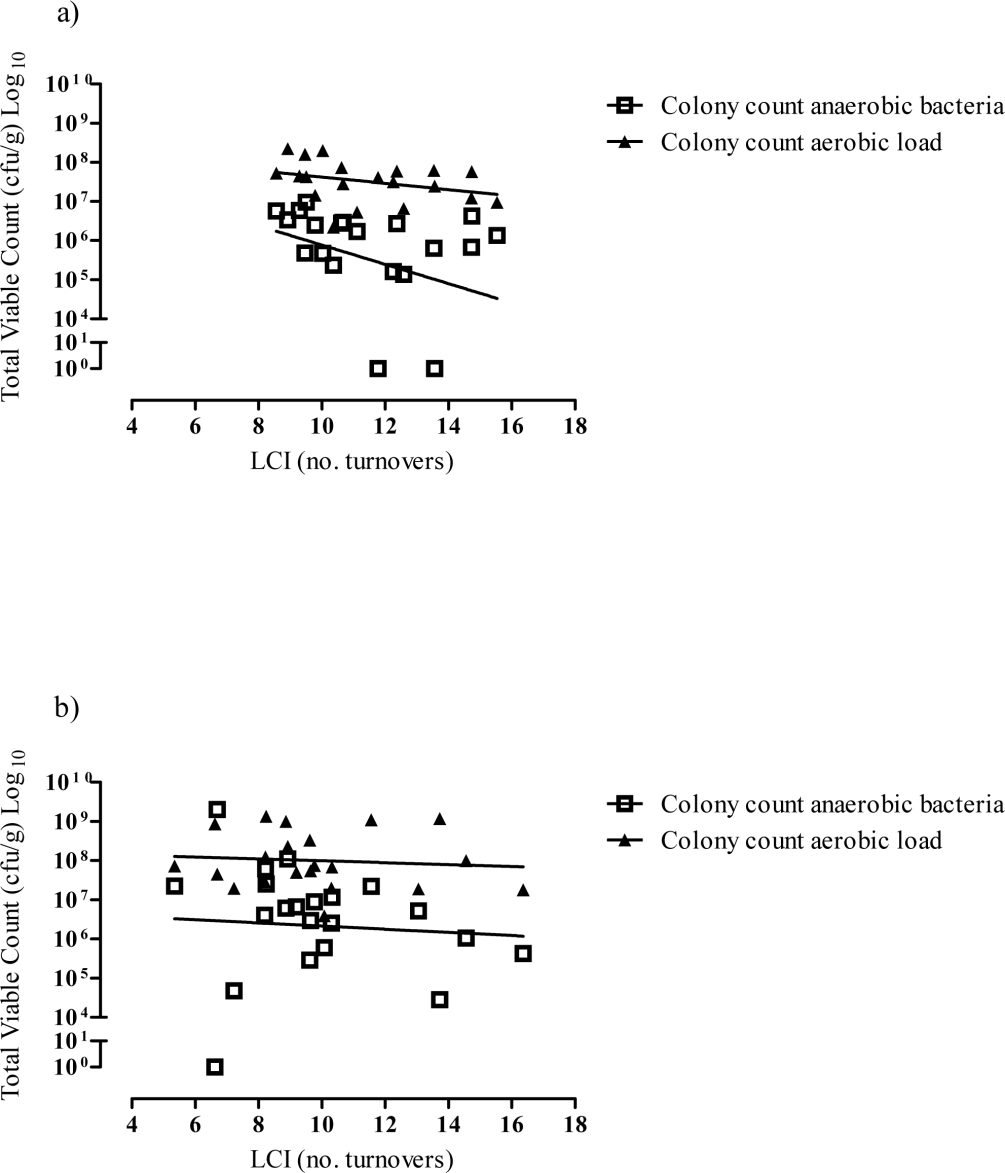
Relationship between anaerobic and aerobic colony count and LCI in CF patients with a) chronic *P. aeruginosa* infection and b) without *P*. *aeruginosa* infection.

Twenty of 41 (49%) patients were chronically colonised with *P*. *aeruginosa*; these patients had a significantly lower colony count of both aerobic (p = 0.03) (including *P*. *aeruginosa*) and anaerobic bacteria (p = 0.04) compared to patients not chronically colonised. The relationship between LCI and colony count of both anaerobic (r = -0.43, p = 0.06) and aerobic bacteria (r = -0.38, p = 0.10) were of moderate strength but not statistically significant in this cohort (Fig 4A).

In the 21/41 (51%) patients without chronic *P*. *aeruginosa* infection, the relationship between LCI and colony count of anaerobic bacteria was not significant (r = -0.29; p = 0.21), heavily influenced by one outlier value (Fig 4B). When this outlier was excluded, an inverse correlation was observed (r = -0.47, p = 0.04). The relationship between LCI and colony count of aerobic bacteria was not significant (r = -0.12, p = 0.61). There was no relationship between colony count of pseudomonas where present (20/41; 49%) and either LCI (r = 0.04; p = 0.86) or FEV_1_ z-score (r = -0.23; p = 0.36).

### Impact of antibiotics on microbial colony counts

There was no significant difference in the colony count of aerobic (p = 0.16) or anaerobic bacteria (p = 0.06) between patients on anti-pseudomonal treatment compared with patients not on treatment. There was also no significant difference in the colony count of aerobic (p = 0.22) or anaerobic bacteria (p = 0.15) between patients on azithromycin treatment compared with patients not on treatment. Using a regression model, neither anti-pseudomonal (beta = -0.11; p = 0.55) nor azithromycin treatment (beta = -0.04; p = 0.85) were shown to be significant predictors of anaerobic colony count.

### Relationship between CRP and microbial colony counts

**Fig 5:**
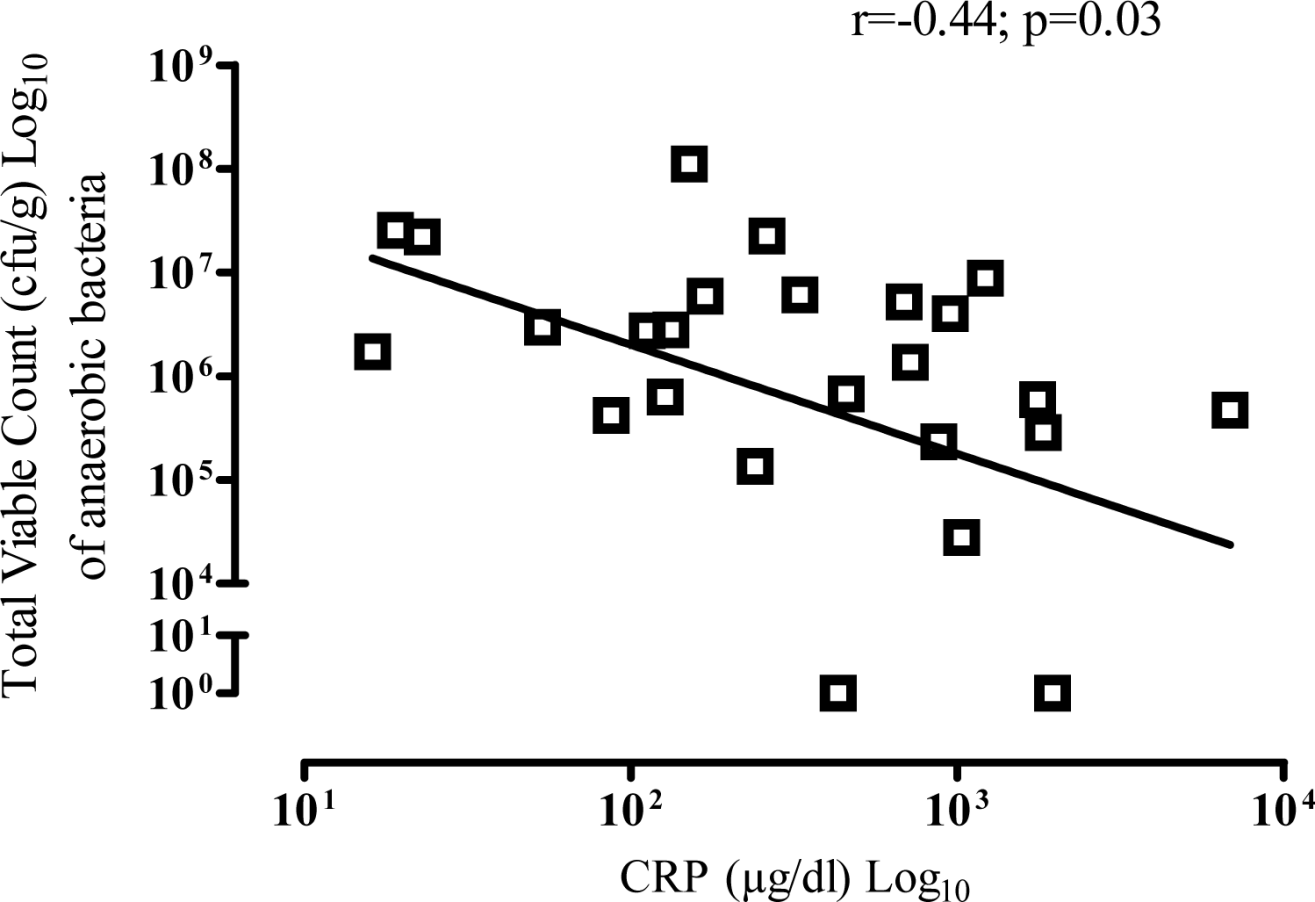
Relationship between colony count of anaerobic bacteria and CRP.

Blood CRP results were available for 25 CF patients. Patients had a median (IQR) CRP of 330.0 (990.4.0–119.8) μg/dl. There was an inverse correlation between colony count of anaerobic bacteria and CRP (r = -0.44; p = 0.03) (Fig 5). However, the correlation between colony count of aerobic bacteria and CRP (r = -0.05; p = 0.82) was not significant.

## Discussion

This is the first study to examine the relationship between colony count of aerobic and anaerobic bacteria, lung disease severity as measured by LCI and spirometry, and inflammation, in a clinically stable group of children and adults with CF. Results of this study demonstrate important relationships between aerobic and anaerobic bacteria and LCI, and between anaerobic bacteria and inflammation.

Extended aerobic and anaerobic culture of CF samples revealed the presence of complex polymicrobial communities with a high abundance of aerobic and anaerobic genera previously detected in studies by this group and others [6,7]. Results from sample analysis of healthy controls also reveal a diverse airway microbiota with similarities in some of the aerobic (*Gemella*, *Staphylococcus)* and anaerobic (*Prevotella*, *Veillonella*) bacteria detected, demonstrating that these genera reside in healthy lungs in the presence of normal LCI and FEV_1_ values. Furthermore, this suggests that these anaerobic bacteria may represent part of the normal flora of the airway microbiota, as shown by other studies [26–28].

Within the CF group, patients with a higher colony count of aerobic and anaerobic bacteria had a better LCI, indicating better lung function. In relation to anaerobes, results from a previous study by Zemanick et al. support these findings, where the presence of anaerobes determined by pyrosequenecing at the time of a PEx was associated with a better lung function as measured by FEV_1_% predicted, compared with the presence of *Pseudomonas*. Relative abundance of *Prevotella* also inversely correlated with serum CRP [11]. The predominant anaerobic genera detected in CF samples in this study (*Prevotella*, *Veillonella*) have been recognised as members of the “core” group in the lung microbiota in CF [29]. Worse LCI in patients with a lower colony count of anaerobic bacteria, may reflect disruption in the composition of the core microbiota and lung disease progression, as has been suggested in COPD [12,28]. The significant inverse correlation between colony count of anaerobic bacteria and CRP values further supports the hypothesis that a decrease in colony count of anaerobic bacteria may be associated with more inflammation and possibly disease progression.

There was also a significant inverse correlation between colony count of aerobic bacteria and LCI. This result was not expected, given that several studies have shown that increased aerobic pathogen colony count is associated with worse clinical outcomes in CF [5,30]. However, this finding may be explained by the fact that, in the present study, the colony count of aerobic bacteria contained both typical CF pathogens and aerobic bacteria recently identified as members of the CF airway microbiota such as *Rothia*, *Gemella* and *Neisseria*.

The relationship between *P*. *aeruginosa* infection and lung function as measured by spirometry and LCI is well established [1,17]. A number of studies have also reported decreased diversity in the lung microbiota in patients with chronic *P*. *aeruginosa* infection, in association with worse lung function [4,11]. Findings from this study of significantly lower counts of anaerobic bacteria, in patients with chronic *P*. *aeruginosa* infection, provide further evidence of an association between low diversity and dominance with *P*. *aeruginosa* infection. However, (excluding the outlier) the relationship between LCI and colony count of anaerobic bacteria was only significant in those patients without *P*. *aeruginosa* infection. This result highlights that the association between decreasing diversity and lung function may also be important to consider in patients without *P*. *aeruginosa* infection.

The use of extended aerobic and anaerobic culture techniques in this study was advantageous as it enabled the detection of organisms of interest and a quantitative estimation of their bacterial load. However, pyrosequencing methods may have enabled a more comprehensive characterisation of airway microbial community composition and provided further evidence of a relationship between diversity and lung disease severity.

These findings highlight the improved sensitivity of LCI compared with FEV_1_ z-score, which did not detect any significant relationships with colony counts. As LCI is representative of disease processes in the peripheral airways, to which FEV_1_ is insensitive, it may provide a more sensitive measure of the impact of infection on the airways. This has been demonstrated in a study of known CF pathogens (*P*. *aeruginosa*, *S*. *aureus*), where LCI was a more sensitive discriminator between infection type, compared with FEV_1_% predicted [16]. Although there was no significant relationship between small airways function, as measured by FEF_25–75_ z-score, and colony counts, the variation within the measure mean can be unreliable. A body of evidence now exists to demonstrate the superior sensitivity of LCI compared with FEV_1_ in younger CF patients with mild lung disease [14]. Our study demonstrates that LCI is also sensitive in older patients with more severe lung disease, as well as younger patients with milder disease with superior discriminatory power compared with FEV_1_ z-score.

Although the difference in anaerobic colony count between patients taking anti-pseudomonal treatment versus patients not taking treatment was not statistically significant, the median difference (4.0 x 10^6^ cfu/g) trended towards a lower count in those taking treatment (p = 0.06). Although treatment was not shown to be a significant predictor of anaerobic colony count in this study, other studies have highlighted antibiotic treatment as a primary driver for changes in microbiota community structure [31] and should be considered in future studies.

This study has a number of limitations including the small number of healthy control subjects analysed (n = 6) due to inadequate volumes of induced sputum produced. This was also a challenge in CF patients where 21/63 (33%) patients did not expectorate adequate sputum for culture analysis. These CF patients were likely to be less symptomatic with better lung function and these results may not extrapolate to this patient group. Although the CF group studied had a wide age range (8–67), the majority were older (mean 29.2 years), potentially impacting on the applicability of these results to a younger age group. Finally, these data are from a single time point and further study of larger numbers over longitudinal time points are required to confirm these relationships.

The authors acknowledge that the relationships demonstrated between LCI and colony counts, and between colony counts and CRP were only of moderate strength (r = -0.44). The relationship between the presence of anaerobic bacteria and lung disease severity may be affected by the presence of other aerobic or anaerobic bacteria. A number of studies have demonstrated that the presence of polymicrobial infection can lead to synergistic interactions, affecting the pathogenicity of aerobic and anaerobic bacteria within the lung [32,33]. Furthermore, infectious processes may also be affected by interactions between microbes and the host. Therefore, the strength of relationship between disease severity and anaerobes may be influenced by the specific airway microbiota composition in each individual.

## Conclusions

This is the first study to show that a lower colony count of aerobic and anaerobic bacteria correlated with a worse LCI. A lower colony count of anaerobic bacteria also correlated with higher CRP levels. These results indicate that lower abundance of aerobic and anaerobic bacteria may reflect microbiota disruption and disease progression in the CF lung.

## Supporting Information

S1 Fig Recruitment flowchart of a) CF patients and b) Control subjects. (PDF)

S2 Fig Relationship between total viable count of *Veillonella* and a) LCI and b) FEV_1_ z-score. (PDF)

S1 File Culture and subsequent detection of isolates in sputum and induced samples. (DOCX)

S2 File Standard operating procedure for Multiple Breath Washout (MBW) testing to measure Lung Clearance Index (LCI). (DOCX)

## References

[1] Konstan MW, Wagener JS, Vandevanter DR, Pasta DJ, Yegin A, Rasouliyan L, et al Risk factors for rate of decline in FEV_1_ in adults with cystic fibrosis. J Cyst Fibros. 2012;11: 405–411. 10.1016/j.jcf.2012.03.009 22561369 PMC4086189

[2] Dasenbrook EC, Checkley W, Merlo CA, Konstan MW, Lechtzin N, Boyle MP. Association between respiratory tract methicillin-resistant *Staphylococcus aureus* and survival in cystic fibrosis. JAMA. 2010;303: 2386–2392. 10.1001/jama.2010.791 20551409

[3] Downey DG, Martin SL, Dempster M, Moore JE, Keogan MT, Starcher B, et al The relationship of clinical and inflammatory markers to outcome in stable patients with cystic fibrosis. Pediatr Pulmonol. 2007;42: 216–220. 17238189 10.1002/ppul.20553

[4] Cox MJ, Allgaier M, Taylor B, Baek MS, Huang YJ, Daly RA, et al Airway microbiota and pathogen abundance in age-stratified cystic fibrosis patients. PLoS ONE. 2010;5: e11044 10.1371/journal.pone.0011044 20585638 PMC2890402

[5] Tunney MM, Klem ER, Fodor AA, Gilpin DF, Moriarty TF, McGrath SJ, et al Use of culture and molecular analysis to determine the effect of antibiotic treatment on microbial community diversity and abundance during exacerbation in patients with cystic fibrosis. Thorax. 2011;66: 579–584. 10.1136/thx.2010.137281 21270069 PMC12747715

[6] Tunney MM, Field TR, Moriarty TF, Patrick S, Doering G, Muhlebach MS, et al Detection of anaerobic bacteria in high numbers in sputum from patients with cystic fibrosis. Am J Respir Crit Care Med. 2008;177: 995–1001. 10.1164/rccm.200708-1151OC 18263800

[7] Delhaes L, Monchy S, Frealle E, Hubans C, Salleron J, Leroy S, et al The airway microbiota in cystic fibrosis: A complex fungal and bacterial community—implications for therapeutic management. PLoS One. 2012;7: e36313 10.1371/journal.pone.0036313 22558432 PMC3338676

[8] Parkins MD, Rendall JC, Elborn JS. Incidence and risk factors for pulmonary exacerbation treatment failures in patients with cystic fibrosis chronically infected with pseudomonas aeruginosa. Chest. 2012;141: 485–493. 10.1378/chest.11-0917 21835906

[9] Armstrong DS, Hook SM, Jamsen KM, Nixon GM, Carzino R, Carlin JB, et al Lower airway inflammation in infants with cystic fibrosis detected by newborn screening. Pediatr Pulmonol. 2005;40: 500–510. 16208679 10.1002/ppul.20294

[10] Sherrard LJ, Graham KA, McGrath SJ, McIlreavey L, Hatch J, Muhlebach MS, et al Antibiotic resistance in prevotella species isolated from patients with cystic fibrosis. Journal of Antimicrobial Chemotherapy. 2013;68: 2369–2374. 10.1093/jac/dkt191 23696621 PMC3772740

[11] Zemanick ET, Harris JK, Wagner BD, Robertson CE, Sagel SD, Stevens MJ, et al Inflammation and airway microbiota during cystic fibrosis pulmonary exacerbations. PLoS One. 2013;8: e62917 10.1371/journal.pone.0062917 23646159 PMC3639911

[12] Erb-Downward J, Thompson DL, Han MK, Freeman CM, McCloskey L, Schmidt LA, et al Analysis of the lung microbiome in the Healthy Smoker and in COPD. PLoS ONE. 2011;6: e16384 10.1371/journal.pone.0016384 21364979 PMC3043049

[13] Hilty M, Burke C, Pedro H, Cardenas P, Bush A, Bossley C, et al Disordered microbial communities in asthmatic airways. PLoS One. 2010;5: e8578 10.1371/journal.pone.0008578 20052417 PMC2798952

[14] Kent L, Reix P, Innes JA, Zielen S, Le Bourgeois M, Braggion C, et al Lung clearance index: Evidence for use in clinical trials in cystic fibrosis. J Cyst Fibros. 2014;13: 123–138. 10.1016/j.jcf.2013.09.005 24315208

[15] Horsley AR, Gustafsson PM, Macleod KA, Saunders C, Greening AP, Porteous DJ, et al Lung clearance index is a sensitive, repeatable and practical measure of airways disease in adults with cystic fibrosis. Thorax. 2008;63: 135–140. 17675315 10.1136/thx.2007.082628

[16] Kraemer R, Baldwin DN, Ammann RA, Frey U, Gallati S. Progression of pulmonary hyperinflation and trapped gas associated with genetic and environmental factors in children with cystic fibrosis. Respir Res. 2006;7: 138 17137500 10.1186/1465-9921-7-138 PMC1698484

[17] Aurora P, Gustafsson P, Bush A, Lindblad A, Oliver C, Wallis CE, et al Multiple breath inert gas washout as a measure of ventilation distribution in children with cystic fibrosis. Thorax. 2004;59: 1068–1073. 15563707 10.1136/thx.2004.022590 PMC1746917

[18] Vermeulen F, Proesmans M, Boon M, Havermans T, De Boeck K. Lung clearance index predicts pulmonary exacerbations in young patients with cystic fibrosis. Thorax. 2014;69: 39–45. 10.1136/thoraxjnl-2013-203807 24021874

[19] Rosenstein BJ, Cutting GR. The diagnosis of cystic fibrosis: A consensus statement. J Pediatr. 1998;132: 589–595. 9580754 10.1016/s0022-3476(98)70344-0

[20] Lee TWR, Brownlee KG, Conway SP, Denton M, Littlewood JM. Evaluation of a new definition for chronic pseudomonas aeruginosa infection in cystic fibrosis patients. Journal of Cystic Fibrosis. 2003;2: 29–34. 15463843 10.1016/S1569-1993(02)00141-8

[21] Hannon D, Bradley JM, Bradbury I, Bell N, Elborn JS, O'Neill K. Shortened lung clearance index is a repeatable and sensitive test in children and adults with cystic fibrosis. BMJ Open Respiratory Research. 2014;1. 10.1136/bmjresp-2014-000031 PMC4212720 25478180

[22] Davies J, Sheridan H, Bell N, Cunningham S, Davis SD, Elborn JS, et al Assessment of clinical response to ivacaftor with lung clearance index in cystic fibrosis patients with a G551D-CFTR mutation and preserved spirometry: A randomised controlled trial. The Lancet Respiratory Medicine. 2013;1: 630–638. 10.1016/S2213-2600(13)70182-6 24461666

[23] Horsley AR, Davies JC, Gray RD, Macleod KA, Donovan J, Aziz ZA, et al Changes in physiological, functional and structural markers of cystic fibrosis lung disease with treatment of a pulmonary exacerbation. Thorax. 2013;68: 532–539. 10.1136/thoraxjnl-2012-202538 23396354

[24] Miller MR, Hankinson J, Brusasco V, Burgos F, Casaburi R, Coates A, et al Standardisation of spirometry. European Respiratory Journal. 2005;26: 319–338. 16055882 10.1183/09031936.05.00034805

[25] Stanojevic S, Wade A, Stocks J, Hankinson J, Coates AL, Pan H, et al Reference ranges for spirometry across all ages. American Journal of Respiratory and Critical Care Medicine. 2008;177: 253–260. 18006882 10.1164/rccm.200708-1248OC PMC2643211

[26] Charlson ES, Bittinger K, Haas AR, Fitzgerald AS, Frank I, Yadav A, et al Topographical continuity of bacterial populations in the healthy human respiratory tract. Am J Respir Crit Care Med. 2011;184: 957–963. 10.1164/rccm.201104-0655OC 21680950 PMC3208663

[27] Morris A, Beck JM, Schloss PD, Campbell TB, Crothers K, Curtis JL, et al Comparison of the respiratory microbiome in healthy nonsmokers and smokers. Am J Respir Crit Care Med. 2013;187: 1067–1075. 10.1164/rccm.201210-1913OC 23491408 PMC3734620

[28] Zakharkina T, Heinzel E, Koczulla RA, Greulich T, Rentz K, Pauling JK, et al Analysis of the airway microbiota of healthy individuals and patients with chronic obstructive pulmonary disease by T-RFLP and clone sequencing. PLoS One. 2013;8: e68302 10.1371/journal.pone.0068302 23874580 PMC3706416

[29] van der Gast C,J., Walker AW, Stressmann FA, Rogers GB, Scott P, Daniels TW, et al Partitioning core and satellite taxa from within cystic fibrosis lung bacterial communities. ISME J. 2011;5: 780–791. 10.1038/ismej.2010.175 21151003 PMC3105771

[30] Belessis Y, Dixon B, Hawkins G, Pereira J, Peat J, MacDonald R, et al Early cystic fibrosis lung disease detected by bronchoalveolar lavage and lung clearance index. American journal of respiratory and critical care medicine. 2012;185: 862–873. 10.1164/rccm.201109-1631OC 22323305

[31] Zhao J, Schloss PD, Kalikin LM, Carmody LA, Foster BK, Petrosino JF, et al Decade-long bacterial community dynamics in cystic fibrosis airways. Proceedings of the National Academy of Sciences. 2012;109: 5809–5814. 10.1073/pnas.1120577109 22451929 PMC3326496

[32] Guss AM, Roeselers G, Newton IL, Young CR, Klepac-Ceraj V, Lory S, et al Phylogenetic and metabolic diversity of bacteria associated with cystic fibrosis. ISME J. 2011;5: 20–29. 10.1038/ismej.2010.88 20631810 PMC3105664

[33] Ulrich M, Beer I, Braitmaier P, Dierkes M, Kummer F, Krismer B, et al Relative contribution of prevotella intermedia and pseudomonas aeruginosa to lung pathology in airways of patients with cystic fibrosis. Thorax. 2010;65: 978–984. 10.1136/thx.2010.137745 20880875

