# Supplementary figures and images for: Reduced Bacterial Colony Count of Anaerobic Bacteria Is Associated with a Worsening in Lung Clearance Index and Inflammation in Cystic Fibrosis
Source: PLoS One. 2015 May 20;10(5):e0126980. doi: 10.1371/journal.pone.0126980 (PMC4439045; doi:10.1371/journal.pone.0126980)

**S1 Fig. Recruitment flowchart of a) CF patients and b) Control subjects**

a)

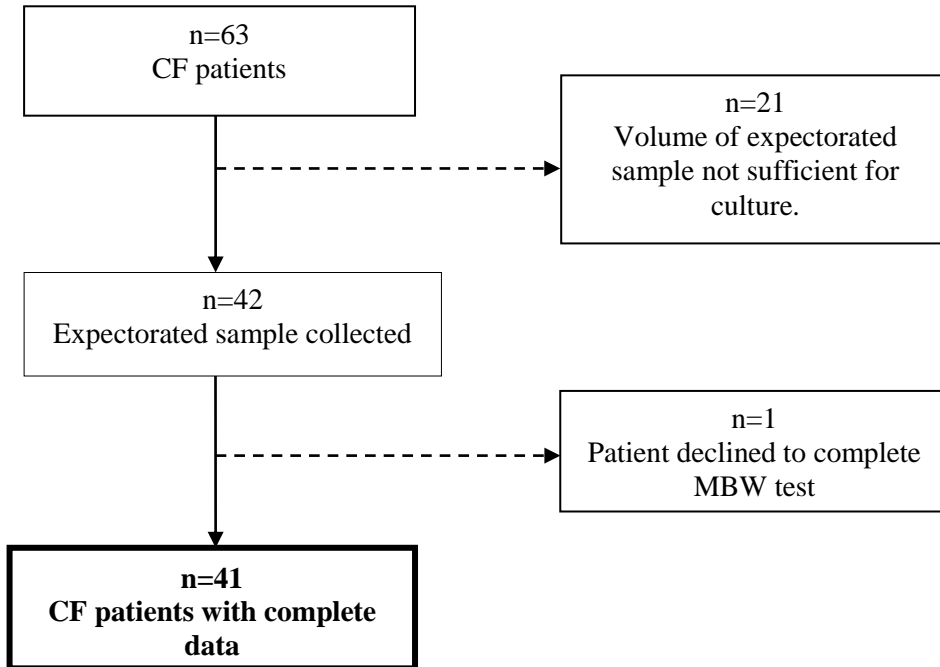

b)

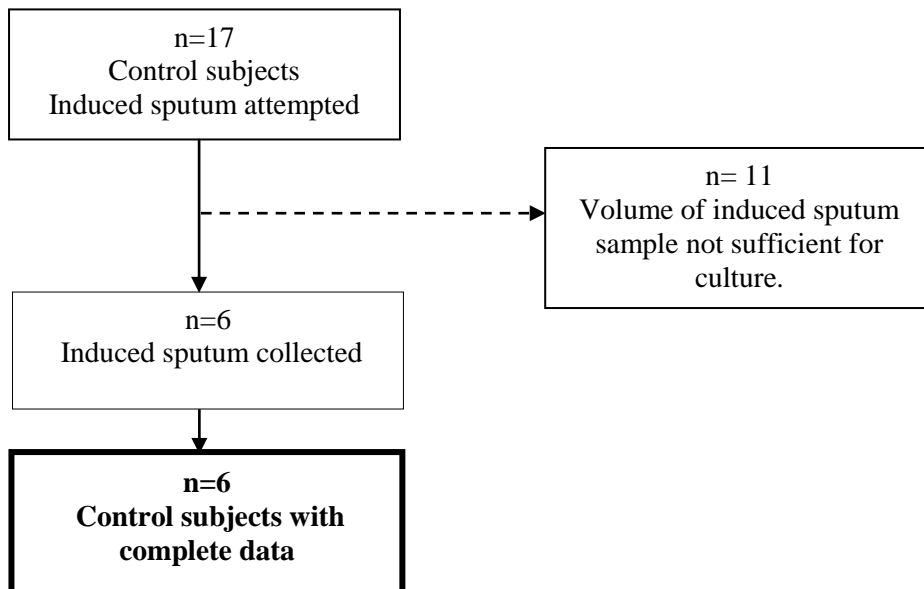

Supplement: S1 Fig — (PDF) [file pone.0126980.s001.pdf]

**S2 Fig. Relationship between total viable count of *Veillonella* and a) LCI and b) FEV<sub>1</sub> z-score.**

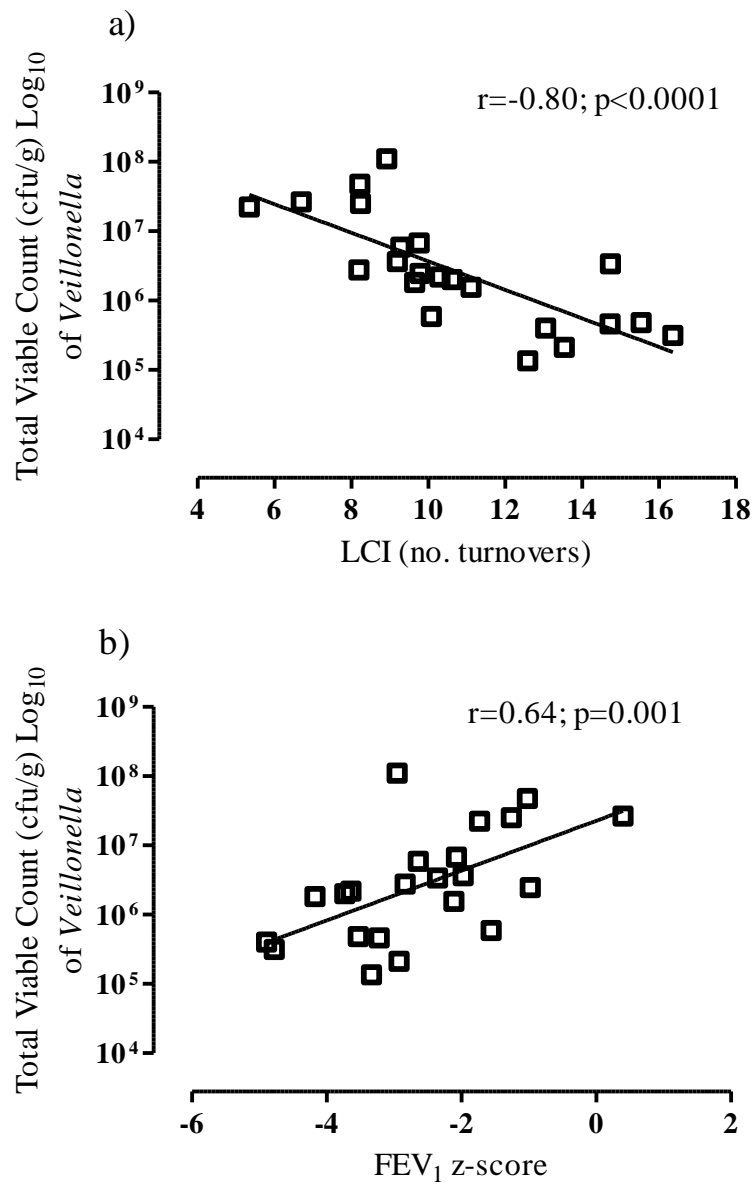

Supplement: S2 Fig — (PDF) [file pone.0126980.s002.pdf]
