# Supplementary material for: Reduced Bacterial Colony Count of Anaerobic Bacteria Is Associated with a Worsening in Lung Clearance Index and Inflammation in Cystic Fibrosis
Source: PLoS One. 2015 May 20;10(5):e0126980. doi: 10.1371/journal.pone.0126980 (PMC4439045; doi:10.1371/journal.pone.0126980)
Supplement: S1 File — (DOCX) [file pone.0126980.s003.docx]

**S1 File. Culture and subsequent detection of isolates in sputum and induced samples**

**Bacterial isolation and identification: quantitative microbiology**

Induced sputum samples were inspected and sputum plugs selected. Thereafter the sample was treated as a sputum sample. Sputum samples were pre-treated for 15 minutes with Sputolysin (Calbiochem, La Jolla, CA) in accordance with the manufacturer’s instructions. In brief, Sputolysin was mixed with sputum in a 1:1 (weight:volume) ratio and vortexed intermittently for 15 minutes at 37 ºC. A 1 mL sample of the treated sputum was serially diluted 10-fold in quarter-strength Ringers Lactate (Oxoid, Basingstoke, United Kindgom). One hundred μl aliquots were spread plated onto anaerobic blood agar (ABA, Oxoid, Basingstoke, UK), kanamycin-vancomycin laked blood agar (KVLB), blood chocolate agar (BCA, Oxoid, Basingstoke, UK) and McKay agar. ABA and KVLB plates were incubated anaerobically for 5-7 days at 37ºC. ABA plates were also incubated in atmospheric O_2_. BCA, ABA and McKay plates were also incubated in 5% CO_2_. Following incubation the total viable counts of each distinct colony type were determined and single colonies of each distinct colony type streaked in duplicate onto the same plate as it was isolated from. One plate was incubated at 37ºC anaerobically for 5 days and the other was incubated at 37ºC aerobically for 5 days to check isolates for oxygen sensitivity. Obligate anaerobes were defined as those isolates capable of growing when incubated anaerobically but not when incubated aerobically. Plates were incubated at 37ºC aerobically for 2 days and following incubation the total viable counts of each distinct colony type were determined and single colonies of each distinct colony type streaked onto their respective plates and incubated at 37ºC for 2 days. All isolates were then stored at -70ºC. Anaerobes were stored in 1ml of defibrinated horse blood, gram negative aerobes were stored in 10% glycerol and gram positive aerobes were stored in protect preservation beads (Technical Service Consultants Ltd, Haywood, UK).

**Molecular identification methods**

**Genomic DNA isolation and PCR Reactions:** Genomic DNA was isolated from bacterial cultures freshly grown on agar plates. Genomic DNA from aerobes was isolated using a DNeasy Blood and Tissue kit (Qiagen,Manchester, UK) in accordance with manufacturer’s instructions. Genomic DNA from anaerobes was isolated using a ZR Fecal DNA MiniPrep kit (Zymo Research) in accordance with manufacturer’s instructions.

Each 25μl reaction consisted of 12.5μL MyTaq Red 2x mix (Bioline), 0.2μM of each primer, 11 μL DEPC treated water (Life Technologies, Paisley, UK), 1 μL template DNA. PCR was performed using a Verti® 96-Well Thermal Cycler (Life Technologies, Paisley, UK) and the reaction consisted of an initial 3 minute denaturation at 95°C followed by 30 cycles of denaturation (95°C/20 seconds), annealing (55°C/20 seconds) and extension (72°C/90 seconds). A final extension step at 72°C for 10 minutes was included upon completion of the cycles. PCR products obtained were confirmed as being of the expected size after separation by agarose electrophoresis.

**Gel Electrophoresis:** PCR amplicons were visualised using horitzontal agarose gel electrophoresis on an HE33 mini horizontal submarine unit (Amersham, Buckinghamshire, UK). Gels were 1.5% (w/v) electrophoresis grade agarose (Life Technologies, Paisley, UK) in 1x Tris acetate EDTA (TAE) buffer (Life Technologies, Paisley, UK) containing 0.5μg/mL ethidium bromide (Sigma, Dorset, United Kingdom). Samples were loaded into the gel alongside 3µL Tracklt™ 100 bp DNA Ladder (Life Technologies, Paisley, UK). Electrophoresis was continued for approximately 30 minutes at 4V/cm2 (approx 100V) using a Bio-Rad Basic PowerPack (Bio-Rad, Bath, UK). Presence of PCR products of the expected size were visualised using a UV transilluminator (Gel-Doc®,Bio-Rad, Bath, UK).

**16S Ribosomal DNA Sequence Analysis:** Prior to sequencing, PCR amplicons were purified using ExoSAP (Affymetrix) as per manufacturer’s instructions. Concentration of the PCR amplicons where determined using the band intensity of the gels. Bands were graded as being of high, intermediate and weak intensity and diluted accordingly with DEPC water (Life Technologies, Paisley, UK ). PCR amplions were sent to GENEWIZ^©^ (GENEWIZ Inc, South Plainfields, NJ) to be sequenced in accordance with sample submission guidelines. Nucleotide sequences were compared with previously published sequences using a basic local alignment sequence tool (BLAST, http://blast.ncbi.nlm.nih.gov/Blast.cgi) and RDP (http://rdp.cme.msu.edu/).
