# Supplementary material for: Reduced Bacterial Colony Count of Anaerobic Bacteria Is Associated with a Worsening in Lung Clearance Index and Inflammation in Cystic Fibrosis
Source: PLoS One. 2015 May 20;10(5):e0126980. doi: 10.1371/journal.pone.0126980 (PMC4439045; doi:10.1371/journal.pone.0126980)
Supplement: S2 File — (DOCX) [file pone.0126980.s004.docx]

**S2 File. Standard operating procedure for Multiple Breath Washout (MBW) testing to measure Lung Clearance Index (LCI).**

The Multiple Breath Washout (MBW) test to measure Lung Clearance Index (LCI) values was carried out using the modified Innocor^TM^ device and 0.2% sulfur hexafluoride (SF_6_) using the open-circuit technique in accordance with the standard operating procedure developed by the UK CF Gene Therapy Consortium (UKCFGTC).

**Equipment setup**

A summary of the Innocor^TM^ equipment set up, in accordance the ERS/ATS consensus statement on MBW measurement is described. ^1^ The Innocor^TM^ equipment set up is described in detail in the original validation paper. ^2^

Before each test, the flowmeter was linearised and calibrated. The flow gas delay (FGD) was calculated. During the test, the Innocor^TM^ software provides visual display of gas concentration, flow and tidal volume allowing identification of the end of wash-in and washout phases and any immediate technical issues.

The modified Innocor^TM^ device meets a number of key recommendations as outlined in the ERS/ATS IGW measurement consensus statement. [1] These include:

- Accurate flow measurement within 1% across a range of flows.
- No volume drift in stable gas concentrations.
- Gas analyser accuracy with excellent signal quality.
- Data sampling frequency of 100Hz.
- Accurate synchronisation of gas and flow signals.^2^

## Performing the test

The test was performed either before or at least 30 minutes after performing spirometry to avoid any effects from a recent forced expiratory manoeuvre. The patient was seated, wearing a noseclip, and asked to breathe normally. Quiet tidal breathing was encouraged by distraction by watching an age-appropriate DVD. The investigator monitored the Innocor^TM^ online display of flow, breath volume, minute volume and gas concentration to ensure a steady breathing pattern. During the wash-in phase the flowmeter was connected to the flowpast T-piece and the subject breathed a mixture of 0.2% SF_6_ in dry air, continuing until the Innocor^TM^ online display indicated that the difference between end-inspiration and end-expiration SF_6_ was 0.003% or less for three successive breaths. The flowpast T-piece was disconnected from the flowmeter early in expiration during a subsequent breath and the flowpast shut off, the washout phase beginning with the next inspiration. Washout continued until the maximum expired concentration of SF_6_ on the Innocor online display was 0.003% or less for three successive breaths. At least three MBW tests were performed at each visit.

## Analysis of MBW data

Innocor^TM^ generates raw flow and gas concentration data. These files were retrieved and transferred via a USB drive onto a PC and converted to text files using software developed by the manufacturer (Innofileconvertor). These text files were then uploaded into the Simple Washout (SW) programme written in the data analysis package Igor Pro, developed by Dr. Nick Bell (UKCFGTC) and used with his permission. The SW programme was used to calculate FRC and subsequently LCI.

Height and the FGD was entered manually to allow for analysis. The programme displays the full wash-in and washout, with flow (L/s) and SF_6_ concentration (%) signals over time (s), re-aligned according to flow gas delay. Breath volume is derived from integration of the flow, and total SF_6_ volume is derived from the integration of flow and gas concentration.

Each test was individually assessed for validity using quality criteria as facilitated by the SW programme. Quality criteria were as follows:

- Good quality disconnection: Ensuring disconnection during expiration with no leaks, re-breathing or irregular descent of SF_6_.
- Complete wash-in: Complete wash-in was defined as a concentration of SF_6_ of ≤0.003% for three consecutive breaths. The SW programme generates a “Cwashin” value (a numerical value of how well washed in the test is), which is determined by SF_6_ concentration at the end of the last inspiration before disconnection minus the concentration of SF_6_ at the end of the last expiration. A “Cwashin” value of ≤ 0.004% was considered ideal; however, values of < 0.008% were considered for inclusion in the calculation of the mean values.

Complete washout: Complete washout is defined to be complete when end tidal SF_6_ concentration falls to ≤1/40^th^ of the starting concentration ^3^ for at least three successive breaths. This equates to an SF_6_ concentration of ≤0.003% for at least three successive breaths.

The FRC was calculated by dividing the total expired volume of SF_6_ over the course of the washout (the last breath of the washout is defined as the first with an end tidal SF_6_ concentration of ≤1/40^th^ of the starting concentration which is followed by two subsequent breaths meeting the same criterion) by [the initial SF6 concentration before disconnection *minus* end tidal expired SF6 concentration at the end of the last breath].

The cumulative expired volume (CEV) is the total volume expired during the washout.

Therefore:

- **LCI = CEV / FRC**

All tests were then checked for repeatability. FRC values between tests should be within 10% of each other and LCI values between tests should be within 20% of each other. Finally, mean FRC and LCI were calculated from a minimum of two valid and repeatable tests.

1. Robinson PD, Latzin P, Verbanck S, et al. Consensus statement for inert gas washout measurement using multiple- and single- breath tests. European Respiratory Journal 2013; 41: 507-522.

2. Horsley AR, Gustafsson PM, Macleod KA, et al. Lung clearance index is a sensitive, repeatable and practical measure of airways disease in adults with cystic fibrosis. Thorax 2008.

3. Bouhuys A and van Lennep HJ. Effect of body posture on gas distribution in the lungs. Journal of Applied Physiology 1962; 17: 38-42.
